# How can we objectively categorise partnership type? A novel classification of population survey data to inform epidemiological research and clinical practice

**DOI:** 10.1136/sextrans-2016-052646

**Published:** 2016-08-17

**Authors:** C H Mercer, K G Jones, A M Johnson, R Lewis, K R Mitchell, K Gravningen, S Clifton, C Tanton, P Sonnenberg, K Wellings, J A Cassell, C S Estcourt

**Affiliations:** 1 Research Department of Infection and Population Health, Centre for Sexual Health and HIV Research, University College London, London, UK; 2 Department of Social and Environmental Health Research, Centre for Sexual and Reproductive Health Research, London School of Hygiene and Tropical Medicine, London, UK; 3 Department of Sociology, University of the Pacific, Stockton, USA; 4 MRC/CSO Social and Public Health Sciences Unit, Institute of Wellbeing, University of Glasgow, Glasgow, UK; 5 Department of Microbiology and Infection Control, University Hospital of North Norway, Tromsø, Norway; 6 Division of Primary Care and Public Health, Brighton and Sussex Medical School, University of Brighton, Brighton, UK; 7 Blizard Institute, Barts and the London School of Medicine and Dentistry, London, UK; 8 Barts Sexual Health Centre, St Bartholomew's Hospital, London, UK

**Keywords:** SEXUAL BEHAVIOUR, CONTACT TRACING, EPIDEMIOLOGY (GENERAL), HEALTH SERV RESEARCH, PARTNER NOTIFICATION

## Abstract

**Background:**

Partnership type is a determinant of STI risk; yet, it is poorly and inconsistently recorded in clinical practice and research. We identify a novel, empirical-based categorisation of partnership type, and examine whether reporting STI diagnoses varies by the resulting typologies.

**Methods:**

Analyses of probability survey data collected from 15 162 people aged 16–74 who participated in Britain's third National Survey of Sexual Attitudes and Lifestyles were undertaken during 2010–2012. Computer-assisted self-interviews asked about participants' ≤3 most recent partners (N=14 322 partners/past year). Analysis of variance and regression tested for differences in partnership duration and perceived likelihood of sex again across 21 ‘partnership progression types’ (PPTs) derived from relationship status at first and most recent sex. Multivariable regression examined the association between reporting STI diagnoses and partnership type(s) net of age and reported partner numbers (all past year).

**Results:**

The 21 PPTs were grouped into four summary types: ‘cohabiting’, ‘now steady’, ‘casual’ and ‘ex-steady’ according to the average duration and likelihood of sex again. 11 combinations of these summary types accounted for 94.5% of all men; 13 combinations accounted for 96.9% of all women. Reporting STI diagnoses varied by partnership-type combination, including after adjusting for age and partner numbers, for example, adjusted OR: 6.03 (95% CI 2.01 to 18.1) for men with two ‘casual’ and one ‘now steady’ partners versus men with one ‘cohabiting’ partner.

**Conclusions:**

This typology provides an objective method for measuring partnership type and demonstrates its importance in understanding STI risk, net of partner numbers. Epidemiological research and clinical practice should use these methods and results to maximise individual and public health benefit.

## Background

Understanding an individual's risk of acquiring a STI requires knowledge not only of the number of sexual partners they have, but also the nature of the relationship, as this influences the likelihood condoms are used and of other risk reduction practices,^1^ and the likelihood that their partner(s) have other partners.^2^ The epidemiological importance of this is reflected in the UK national clinical guidelines, which state that STI risk assessments should ask patients about all partners in the past 3 months, including the nature of their relationships.^3^ This information is also important for partner notification (PN), as it can dictate the method of PN employed and its probability of success.^4–7^ There has thus been a call for PN process outcomes to be measured in terms of *partner*-centred outcomes (eg, transmissions prevented according to partnership type) rather than *patient*-centred outcomes (eg, partners seen per index case) to better measure impact and to determine optimal use of PN resources.^8^ Despite this, in England, the mandatory surveillance system for STI diagnoses (the Genitourinary Medicine Clinic Activity Dataset) does not currently require data to be collected on partnership type;^9^ ^10^ so, monitoring PN in terms of *partner*-centred outcomes remains an aspiration. One likely reason for its absence is the lack of consensus in both professional and lay contexts regarding what constitutes different types of partnership, for example, what differentiates a regular partner from a casual partner?^11^

In this paper, we seek to distinguish between different types of sexual partnership by applying statistical analysis techniques to data on two particular partnership characteristics: (1) partnership duration and (2) perceived likelihood of sex again with the partner. We examine the extent to which the resulting thresholds for different partnership types vary by key sociodemographic characteristics, and by sexual health clinic attendance, recognising the potential use of a typology in this setting, as well as clinic attendees' tendency to report greater sexual risk relative to the general population.^12^ ^13^ We then consider an application of the resulting partnership typology by examining whether, and if so how, reporting STI diagnosis in the past year varies according to recent sexual partnership history in terms of the type(s) of partner individuals have. Given the well-established association between partner numbers and STI diagnosis,^13^ we examine whether any association with partnership type exists after adjusting for age and the number of sexual partners reported.

## Methods

We analysed data collected by the third National Survey of Sexual Attitudes and Lifestyles (Natsal-3), a probability survey of 15 162 people aged 16–74 resident in Britain, undertaken during 2010–2012. Complete details of the methodology of Natsal-3 have been published previously, including the full questionnaire.^14^ ^15^ The Natsal-3 study was approved by the Oxfordshire Research Ethics Committee A (reference: 09/H0604/27). Participants provided oral informed consent for interviews.

Natsal-3 used computer-assisted personal interviewing with computer-assisted self-interview for the most sensitive questions, for example, the number of partners in various timeframes, and a module that asked about participants' most recent partners.^14^ Questions in this module relevant to this study were: date (month/year) of the first and most recent sex with this partner, and whether they thought they would have sex again with the partner. They were also asked, “which one of these descriptions applies best to you [and your partner] at the time you most recently had sex?”: “we were living together as a couple/married/in a civil partnership at the time” (hereon referred to as ‘living together’), “in a steady relationship at the time” (hereon ‘steady’), “we used to be in a steady relationship, but were not at that time” (hereon ‘ex-steady’), “we had known each other for a while, but were not in a steady relationship” (hereon ‘known a while’), “we had recently met” (hereon ‘recently met’) and “we had just met for the first time” (hereon ‘just met’).^12^ If participants reported sex with the partner on more than one occasion, then they were asked a similar question—with the same response options—about the first occasion. Responses to these two questions enabled us to identify 36 possible ‘partnership progression types’ (PPTs), for example, ‘recently met’ at first sex but ‘steady’ at most recent sex. Twelve of the 36 PPTs, corresponding to 129 partnerships, are ignored hereon, as they are illogical, for example, ‘living together’ at first sex but ‘met recently’ at most recent sex.

If participants reported more than one partner in the past 5 years, then the most recent partner question loop was repeated, and the PPT was derived for the second, and if applicable, third, most recent partners. However, in order to present a contemporary picture of partnerships in Britain, only partnerships where the most recent occasion of sex occurred in the year prior to interview for Natsal-3 were included in these analyses. This timeframe has the additional advantages of lower recall bias, and providing a more complete picture of all partnerships, as a larger proportion of the population have had at most three partners in the past year than in the past 5 years (93.7% vs 79.9% of men, respectively; 96.3% vs 87.5% of women, respectively).^15^

### Statistical analysis

The data were transposed to make the unit of analysis initially partnerships rather than participants. We calculated the duration of each partnership in months, as the date of most recent sex minus the date of first sex with the partner. Partnerships involving sex only once were given a duration of zero months. We then used an analysis of variance (ANOVA) and Tukey's Honestly Significant Difference (HSD) test^16^ to explore the feasibility of collapsing the 24 PPTs into a smaller number of summary partnership types if there were no statistically significant differences in partnership duration between PPTs, as we hypothesised that casual partnerships would be shorter in duration than more regular partnerships. As the partnership duration data were highly skewed, the data were rank-transformed before running the ANOVA. Logistic regression was then used to examine whether participants' perceived likelihood of having sex again with the partner, ‘yes’/‘probably’ versus *‘*probably not’/‘no’/‘don't know’, varied *between* but not *within* the summary types identified in the ANOVA. We focused on this variable to differentiate between casual and regular partners as in previous studies.^6^ ^7^

To examine whether the same thresholds for partnership duration used to define the summary partnership types also applied for different genders, age groups, sexual identities and reporting sexual health clinic attendance, we used non-parametric tests (Kruskal-Wallis and Mann-Whitney accordingly), because the data were not normally distributed. Similarly, we used logistic regression to examine whether the likelihood of sex again was consistent across these groups.

We then transposed the partnership-level data to make a participant-level dataset and used binary logistic regression to see whether reporting STI diagnosis in the past year varied by the summary partnership type(s) participants had had in the past year. We used multivariable logistic regression to examine this association after adjusting for the confounding effects of age and the number of partners reported in the past year.

All analyses were done using the survey functions in Stata V.13 (StataCorp. 2013. Stata Statistical Software: Release V.13. College Station, TX: StataCorp LP.) to account for the sample weighting, clustering and stratification within the Natsal-3 sample, and the clustering of partnerships ‘within’ participants for analyses conducted at the partnership-level. Statistical significance was considered as p<0.05 for all analyses.

### Data availability

The Natsal-3 dataset is publicly available from the UK Data Service: https://discover. ukdataservice.ac.uk; SN: 7799; persistent identifier: 10.5255/UKDA-SN-77991-1.

## Results

### Frequency of partnership progression types

Altogether, 11 097 Natsal-3 participants (4659 men) completed the most recent partner module, about a total of 14 322 partnerships in the past year. Of these, 14 193 partnerships had a logical PPT (table 1), and of these, 14 150 partnerships had one of 21 PPTs, each corresponding to ≥30 partnerships upon which analyses hereon are based.

**Table 1:** Ranked frequency distribution of logical* partnership progression types (PPTs) of partnerships in the past year reported in detail by participants in Natsal-3, by gender

	Denominators† (unweighted, weighted) PPT		Partnerships reported by all participants		Partnerships reported by male participants		Partnerships reported by female participants	
			14193, 13486		6172, 7067		8021, 6419	
Rank	Relationship status at first sex	Relationship status at most recent sex	Per cent	(95% CI)	Per cent	(95% CI)	Per cent	(95% CI)
1	Steady	Living together	28.5	(27.5 to 29.5)	24.6	(23.3 to 26.0)	32.7	(31.4 to 34.1)
2	Recently met	Living together	10.2	(9.6 to 10.9)	11.6	(10.6 to 12.7)	8.6	(7.9 to 9.4)
3	Known a while	Living together	10.0	(9.4 to 10.7)	10.7	(9.7 to 11.6)	9.3	(8.6 to 10.1)
4	Known a while	Known a while	9.4	(8.8 to 9.9)	10.1	(9.3 to 10.9)	8.6	(7.9 to 9.3)
5	Living together	Living together	9.3	(8.6 to 9.9)	8.3	(7.4 to 9.2)	10.3	(9.4 to 11.3)
6	Steady	Steady	9.2	(8.8 to 9.7)	8.7	(8.0 to 9.4)	9.9	(9.3 to 10.6)
7	Recently met	Recently met	4.9	(4.5 to 5.3)	6.1	(5.5 to 6.7)	3.6	(3.2 to 4.1)
8	Known a while	Steady	3.9	(3.6 to 4.2)	3.4	(3.0 to 3.9)	4.4	(4.0 to 4.9)
9	Recently met	Steady	3.6	(3.3 to 4.0)	3.7	(3.2 to 4.2)	3.5	(3.1 to 4.0)
10	Just met	Just met	2.7	(2.4 to 3.0)	3.9	(3.5 to 4.5)	1.3	(1.1 to 1.6)
11	Recently met	Known a while	1.8	(1.6 to 2.0)	2.2	(1.8 to 2.6)	1.4	(1.1 to 1.6)
12	Steady	Ex-steady	1.3	(1.1 to 1.5)	1.0	(0.8 to 1.2)	1.7	(1.4 to 2.0)
13	Just met	Living together	1.2	(1.0 to 1.4)	1.3	(1.0 to 1.8)	1.0	(0.7 to 1.3)
14	Known a while	Ex-steady	0.9	(0.7 to 1.0)	0.8	(0.6 to 1.1)	0.9	(0.7 to 1.1)
15	Recently met	Ex-steady	0.9	(0.7 to 1.0)	0.9	(0.7 to 1.1)	0.8	(0.7 to 1.1)
16	Ex-steady	Ex-steady	0.5	(0.4 to 0.7)	0.6	(0.4 to 0.9)	0.4	(0.3 to 0.6)
17	Just met	Steady	0.4	(0.3 to 0.5)	0.4	(0.3 to 0.6)	0.4	(0.3 to 0.6)
18	Just met	Known a while	0.4	(0.3 to 0.5)	0.6	(0.4 to 0.8)	0.2	(0.1 to 0.3)
19	Just met	Recently met	0.4	(0.3 to 0.5)	0.6	(0.4 to 0.8)	0.2	(0.1 to 0.4)
20	Ex-steady	Living together	0.3	(0.2 to 0.4)	0.2	(0.1 to 0.4)	0.4	(0.2 to 0.6)
21	Ex-steady	Steady	0.2	(0.2 to 0.3)	0.2	(0.1 to 0.4)	0.2	(0.1 to 0.3)
	**PPTs with insufficient numbers‡**							
	Living together	Steady	<0.1	(<0.1 to 0.2)	<0.1	(<0.1 to 0.2)	0.1	(<0.1 to 0.3)
	Just met	Ex-steady	<0.1	(<0.0 to 0.1)	<0.1	(<0.1 to 0.2)	<0.1	(<0.1 to 0.1)
	Living together	Ex-steady	<0.1	(<0.1 to 0.1)	<0.1	(<0.1 to 0.1)	<0.1	(<0.1 to 0.1)

*Only logical PPTs are shown in this table; thus excludes the following 12 PPTs:

Living together, known a while; living together, recently met; living together, just met; steady, known a while; steady, recently met; steady, just met; ex-steady, known a while; ex-steady, recently met; ex-steady, just met; known a while, recently met; known a while, just met; recently met, just met. These 12 PPTs correspond to 129 partnerships that are excluded from this table and from further analyses.

†Denominators correspond to the number of partnerships (not participants).

‡<30 such partnerships reported; so, these PPTs and the corresponding partnerships are excluded from further analyses.

The three most common PPTs all involved living together at most recent sex (48.7% of all partnerships, table 1). In the most common PPT, first sex occurred in the context of a steady relationship, and most recent sex while living together (28.5% of all partnerships), and this PPT accounted for a larger proportion of women's partnerships than men's, 32.7% vs 24.6%, respectively. In the next most common PPTs, partners lived together at most recent sex, but first sex occurred when they had recently met (10.2%) or had known a while (10.0%). Around one-third (36.0%) of all partnerships were those in which the relationship status was the same at first and most recent sex, for example, 9.2% of partnerships were described as ‘steady’ on both occasions.

### Identifying summary partnership types

An ANOVA of partnership duration and then logistic regression of the likelihood of having sex again with the partner suggested that all but one of the 21 PPTs can be categorised into one of four ‘summary partnership types’, labelled as ‘cohabiting’, ‘now steady’, ‘casual’ and ‘ex-steady’ (table 2). Cohabitations had the longest median duration of around 171 months (14.25 years) with an IQR of 79–320 months (6.5–26.6 years), and in nearly all of these partnerships (96.8%), the participant anticipated having sex again with this partner. The summary partnership type labelled ‘now steady’ corresponded to much shorter partnerships with a median duration of 15 months (IQR 5–38 months), and about three-quarters of participants anticipated sex again, although this ranged from 60.1% to 83.4%. ‘Casual’ corresponded to partnerships with a median duration of 0 months, meaning that the first and most recent sex had occurred in the same calendar month, which constituted the same event in around half of these partnerships (see online supplementary table S1). While sex again was anticipated in less than one-third of casual partnerships, this ranged from 9.0% to 50.3%. Finally, the summary partnership type of ‘ex-steady’ corresponded to partnerships with a median duration of 38 months (IQR 16–89), and partnership status at most recent sex described as ‘ex-steady’ for all three PPTs. Nonetheless, sex again was anticipated in just under half of these partnerships.

**Table 2:** Average relationship duration and likelihood of sex again by partnership progression type (PPTs), categorised by summary partnership type identified using the Tukey method*

			Duration (months)		Anticipate having sex again	
		Denominators† (unweighted, weighted)	Median (IQR)	Mean (SD)	Per cent	(95% CI)
All partnerships		14048, 13387	73 (10–223)	137.2 (157.5)	79.4	(78.4 to–80.3)
**Summary partnership type: cohabiting**		**6493, 8008**	**171 (79–320)**	**211.2 (160.0)**	**96.8**	(96.4 to 97.2)
Partnership progression type						
Living together	Living together	978, 1247	172 (51–386)	226.4 (194.4)	95.5	(94.0 to 96.6)
Steady	Living together	3047, 3840	228 (108–368)	248.4 (162.3)	97.3	(96.7 to 97.8)
Ex-steady	Living together	32, 37	115 (54–214)	152.1 (123.2)	97.8	(85.6 to 99.7)
Known a while	Living together	1159, 1351	131 (67–232)	161.4 (123.0)	96.9	(95.8 to 97.7)
Recently met	Living together	1138, 1376	126 (61–214)	154.4 (118.9)	96.8	(95.8 to 97.9)
Just met	Living together	139, 158	97 (42–170)	123.8 (107.6)	94.2	(84.8 to 97.9)
**Summary partnership type: now steady**		**3816, 2635**	**15 (5–38)**	**36.3 (61.6)**	**73.3**	**(71.4 to 75.1)**
Partnership progression type						
Steady	Steady	1785, 1247	12 (3–35)	32.8 (60.8)	69.3	(66.5 to 71.9)
Ex-steady	Steady	37, 29	10 (4–20)	18.8 (30.7)	66.9	(48.6 to 81.2)
Known a while	Steady	744, 522	22 (10–55)	46.6 (66.6)	83.4	(79.9 to 86.3)
Recently met	Steady	663, 486	18 (8–40)	35.4 (52.3)	79.7	(75.9 to 83.0)
Recently met	Known a while	311, 240	12 (4–30)	38.2 (75.4)	60.1	(53.4 to 66.4)
Just met	Steady	81, 57	16 (5–41)	39.2 (60.5)	76.2	(64.0 to 85.2)
Just met	Known a while	61, 54	13 (7–22)	21.4 (34.3)	65.8	(50.3 to 78.5)
**Summary partnership type: casual**		**3111, 2339**	**0 (0–2)**	**9.0 (35.1)**	**31.1**	**(29.1** **to** **33.2)**
Partnership progression type						
Known a while	Known a while	1662, 1263	1 (0–10)	14.8 (42.3)	37.7	(34.9 to 40.6)
Recently met	Recently met	817, 662	0 (0–0)	3.2 (27.8)	29.0	(25.5 to 32.9)
Just met	Recently met	66, 53	1 (0–4)	4.2 (13.0)	50.3	(36.7 to 64.0)
Just met	Just met	444, 362	0 (0–0)	0.3 (3.4)	9.0	(6.6 to 12.2)
**Summary partnership type: ex-steady**		**628, 405**	**38 (16–89)**	**70.7 (88.0)**	**45.2**	**(40.5 to 50.0)**
Partnership progression type						
Steady	Ex-steady	270, 174	38 (16–83)	71.3 (92.2)	44.1	(37.3 to 51.1)
Known a while	Ex-steady	167, 117	41 (15–117)	80.1 (98.1)	52.7	(43.3 to 62.0)
Recently met	Ex-steady	149, 114	35 (17–84)	60.1 (86.2)	39.7	(30.3 to 49.0)

*Excludes PPT ‘ex-steady–ex-steady’, as according to the Tukey method, this PPT does not fit within any one particular summary partnership type.

†Denominators correspond to the number of partnerships (not participants).

10.1136/sextrans-2016-052646.supp1 Supplementary tables

### Variation in the thresholds used to define the summary partnership types

There was little gender difference in partnership duration or the likelihood of having sex again for each of the four summary partnership types (table 3), except that women's ex-steady partnerships were longer than men's, on average (median of 43 vs 31 months, respectively). As might be expected, average duration varied by age group for each summary partnership type, as did the likelihood of sex, except for ex-steady partnerships. There was little variation in partnership duration or the likelihood of sex again by sexual identity, although cohabiting partnerships reported by heterosexually identifying participants were longer than those reported by participants identifying as other than heterosexual (median of 174 vs 92 months, respectively). Participants who reported sexual health clinic attendance in the 5 years prior to interview had shorter partnerships, regardless of type, and were less likely to anticipate sex again, except in casual partnerships.

**Table 3:** Extent of variation in partnership duration and likelihood of sex again for each summary partnership type by participant's gender, age group, sexual identity and sexual health clinic attendance

		Duration (months)		Anticipate having sex again	
	Denominators* (unweighted, weighted)	Median (IQR)	Mean (SD)	Per cent	95% CI
***Summary partnership type: cohabiting***	6493, 8008	**171** (**79–320)**	**211.2** (**160.0)**	**96.8**	**(96.4 to 97.2)**
**By participant's gender**		p=0.936		p=0.593	
Male	2594, 4011	164 (76–308)	205.6 (157.6)	96.9	(96.2 to 97.5)
Female	3899, 3998	179 (82–326)	217.0 (162.4)	96.7	(96.1 to 97.2)
**By participant's age group**		p<0.0001		p<0.0001	
16–24	665, 435	33 (18–58)	38.9 (27.1)	92.2	(89.7 to 94.0)
25–34	2268, 1694	79 (43–119)	84.0 (51.4)	96.7	(95.9 to 97.3)
35–44	1327, 2122	156 (93–223)	157.0 (82.7)	97.8	(97.0 to 98.4)
45–54	1014, 1841	271 (160–338)	246.7 (117.4)	97.1	(95.9 to 98.0)
55–64	770, 1264	406 (261–469)	356.7 (148.5)	97.3	(95.9 to 98.2)
65–74	449, 652	520 (357–571)	451.6 (172.2)	95.3	(93.1 to 96.8)
**By participant's sexual identity**		p<0.0001		p=0.066	
Heterosexual	6321, 7829	174 (81–321)	213.0 (160.4)	96.9	(96.4 to 97.3)
Other	158, 160	92 (43–153)	123.7 (115.8)	94.5	(90.4 to 97.0)
**By participant's sexual health clinic attendance**		p<0.0001		p<0.0001	
No	5705, 7360	187 (93–333)	223.6 (159.5)	97.2	(96.8 to 97.6)
Yes, in last 5 years	739, 596	41 (21–82)	68.0 (74.9)	92.1	(89.4 to 94.1)
***Summary partnership type: now steady***	3816, 2635	**15** (**5–38)**	**36.3** (**61.6)**	**73.3**	**(71.4 to 75.1)**
**By participant's gender**		p=0.634		p=0.616	
Male	1616, 1350	16 (5–36)	35.0 (62.4)	73.7	(71.1 to 76.1)
Female	2200, 1285	15 (5–41)	37.3 (60.8)	72.8	(70.6 to 74.9)
**By participant's age group**		p<0.0001		p<0.0001	
16–24	1937, 1186	11 (3–23)	16.1 (18.0)	70.2	(67.7 to 72.5)
25–34	1009, 568	14 (5–37)	29.0 (36.8)	71.3	(68.2 to 74.3)
35–44	291, 289	19 (6–56)	39.1 (49.8)	72.0	(65.6 to 77.7)
45–54	339, 365	38 (11–103)	76.4 (91.9)	80.3	(75.3 to 84.7)
55–64	178, 173	29 (12–83)	78.7 (112.4)	83.1	(76.7 to 88.0)
65–74	61, 55	65 (34–237)	132.4 (131.6)	88.1	(76.3 to 94.4)
**By participant's sexual identity**		p=0.733		p=0.711	
Heterosexual	3619, 2501	15 (5–38)	36.2 (62.3)	73.4	(71.6 to 75.1)
Other	195, 132	19 (5–49)	38.3 (48.2)	72.1	(65.3 to 78.1)
**By participant's sexual health clinic attendance**		p<0.0001		p<0.0001	
No	2474, 1775	17 (5–48)	43.4 (71.3)	75.8	(73.7 to 77.7)
Yes, in last 5 years	1288, 822	12 (4–28)	21.9 (29.1)	68.5	(65.5 to 71.3)
***Summary partnership type: casual***	**3111, 2339**	**0** (**0–2)**	**9.0** (**35.1)**	**31.1**	**(29.1 to 33.2)**
**By participant's gender**		p=0.054		p=0.028	
Male	1660, 1461	0 (0–2)	8.9 (34.7)	32.8	(30.0 to 35.7)
Female	1451, 879	0 (0–2)	9.3 (35.8)	28.3	(25.6 to 31.2)
**By participant's age group**		p<0.0001		p=0.004	
16–24	1590, 1025	0 (0–1)	2.7 (8.9)	28.7	(26.2 to 31.5)
25–34	888, 571	0 (0–2)	5.2 (16.8)	27.5	(23.9 to 31.4)
35–44	282, 326	0 (0–3)	10.2 (33.6)	35.1	(28.3 to 42.5)
45–54	213, 260	0 (0–14)	22.6 (58.1)	34.5	(27.6 to 42.2)
55–64	106, 127	4 (0–24)	38.6 (86.8)	43.4	(32.2 to 55.5)
65–74	32, 30	4 (0–37)	42.6 (79.4)	52.2	(34.4 to 69.4)
**By participant's sexual identity**		p=0.447		p=0.103	
Heterosexual	2893, 2168	0 (0–2)	9.0 (35.2)	30.5	(28.4 to 32.7)
Other	215, 169	0 (0–2)	10.2 (33.6)	37.8	(29.5 to 47.0)
**By participant's sexual health clinic attendance**		p=0.017		p=0.137	
No	1838, 1470	0 (0–3)	12.3 (43.0)	32.2	(29.5 to 35.0)
Yes, in last 5 years	1230, 837	0 (0–2)	3.5 (11.2)	29.2	(26.2 to 32.3)
***Summary partnership type: ex-steady***	628, 405	**38** (**16–89)**	**70.7** (**88.0)**	**45.2**	**(40.5 to 50.0)**
**By participant's gender**		p=0.008		p=0.090	
Male	230, 188	31 (15–84)	68.6 (93.8)	40.6	(33.3 to 48.4)
Female	398, 217	43 (19–95)	72.5 (82.6)	49.3	(43.3 to 55.2)
**By participant's age group**		p<0.0001		p=0.864	
16–24	269, 148	23 (9–40)	27.7 (22.3)	42.8	(36.3 to 49.4)
25–34	228, 127	43 (19–84)	56.2 (46.6)	44.3	(36.7 to 52.1)
35–44	68, 66	103 (23–165)	113.7 (94.2)	48.2	(34.4 to 62.3)
45–54	39, 39	59 (18–154)	102.5 (104.3)	54.0	(35.9 to 71.1)
55–64	21, 22	144 (96–359)	221.5 (150.9)	42.2	(22.1 to 65.2)
65–74	3, 2	340 (340–579)	385.9 (164.0)	39.8	(0.6 to 88.2)
**By participant's sexual identity**		p=0.611		p=0.291	
Heterosexual	591, 379	39 (16–89)	71.5 (88.7)	45.9	(41.1 to 50.8)
Other	37, 26	29 (12–78)	59.5 (76.1)	35.6	(20.3 to 54.5)
**By participant's sexual health clinic attendance**		p=0.005		p=0.002	
No	367, 250	47 (17–1132)	87.5 (103.4)	51.1	(44.9 to 57.3)
Yes, in last 5 years	255, 150	27 (15–56)	42.0 (40.9)	35.6	(28.8 to 43.0)

*Denominators correspond to the number of partnerships (not participants).

### Variation in individuals' reporting of STI diagnosis/es according to partnership type(s)

To examine whether reporting STI diagnosis varied for individuals according to their partnership type(s), it was first necessary to identify all the possible combinations of summary partnership types, as participants could report detailed data on at most three partners. Altogether, 34 combinations were identified (see online supplementary table S2); however, the majority of individuals—around 85%—had one partner in the past year, which was a cohabiting partner for three-quarters of these people. Many of the combinations of summary partnership types represent small proportions and numbers of people; so, we focused on combinations that correspond to at least 50 people. For men, there were 11 such partnership-type combinations, accounting for 94.5% (95% CI 93.6% to 95.3%) of all men; for women, there were 13 such partnership-type combinations, accounting for 96.9% (95% CI 96.5% to 97.3%) of all women.

**Figure 1:**
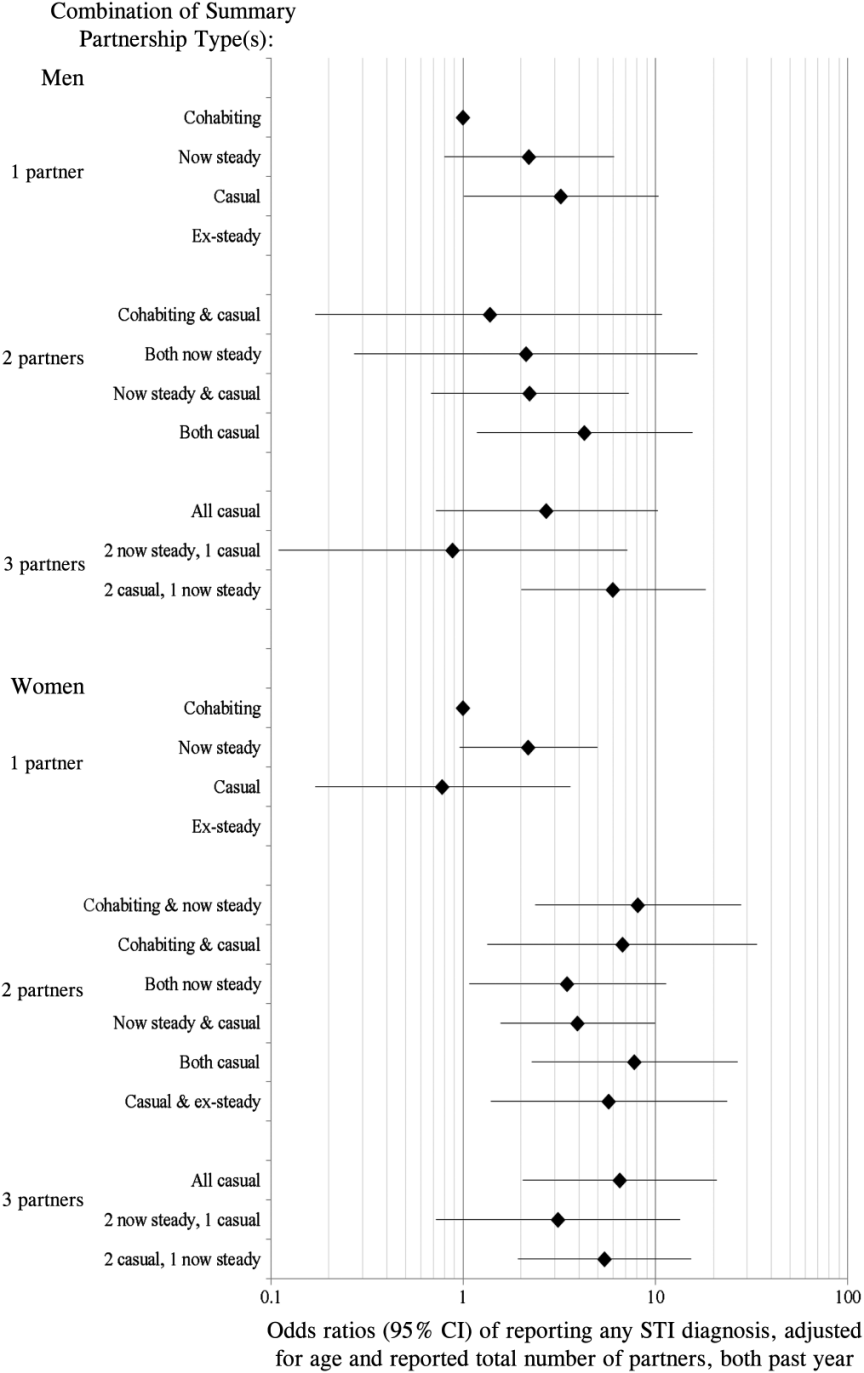
Odds ratios of reporting any STI diagnosis, adjusted for total reported number of partners and age, by combination of Summary Partnership Types, all in the past year, by gender.

The prevalence of reported STI diagnosis in the past year was estimated as 1.0% of all men and women reporting sexual partner(s) during this time. However, this varied considerably according to the combination of partner type(s) men and women had during that time (figure 1, see online supplementary tables S3A,B). Prevalence was lowest among those who reported just one cohabiting partner, at 0.4% (95% CI 0.3% to 0.8%) of men and 0.3% (95% CI 0.2% to 0.5%) of women. It was highest among men whose three most recent partners had been a combination of two casual and one now steady (6.0%, 95% CI 2.5% to 13.5%), and among women whose three most recent partners had all been casual (8.7%, 95% CI 3.8% to 18.9%).

Gender differences in reporting STI diagnosis/es were observed by partnership-type combination. Of note, among those with just one partner, there was no difference for women by whether this was a cohabiting or casual partner, but the OR for men with one casual partner was 4.94 (95% CI 1.67 to 14.6) relative to men with a cohabiting partner. Adjusting for the confounding effect of age and the total number of partners participants reported in the past year reduced the ORs, but marked variations remained according to the combination of partner types men and women had.

## Discussion

Four distinct types of sexual partnership labelled—‘cohabiting’, ‘now steady’, ‘casual’ and ‘ex-steady’—were identified in the British general population from statistical analyses of data collected on partnership duration and the likelihood of having sex again in >14 000 partnerships. Using this typology, we showed that differences exist in reporting STI diagnosis according to the different types of partners people had, which were not explained by differences in the numbers of partners they reported. This emphasises the need for clinical practice and epidemiological research to take account of partnership type to better understand STI risk, and this study provides an objective methodology with which to do so.

Our typology can be considered as broadly representative of partnership types in the British general population because of Natsal-3's sampling strategy. However, as most people in Britain have only one partner in a year, and this is likely to be a partner with whom they live,^15^ the number of people in Natsal-3 reporting more than one partner was small, especially when broken down by partnership type. Our statistical power was further limited by using a timeframe of the past year to define our outcome of reported STI diagnosis. However, this timeframe tallies with our definition of recent partnerships, which we used in order to capture a larger proportion of all partnerships and reduce recall bias, the latter being particularly important as date data were needed to derive the partnership typology. While Natsal-3 collected urine samples for STI testing,^13^ using these data would have reduced our statistical power even further, as biological samples were only collected from a subsample of participants aged 16–44.

The thresholds used to define the four summary partnership types applied in the context of the British population, regardless of gender and sexual identity (although we acknowledge that we considered sexual identity crudely, reflecting the relatively low prevalence of non-heterosexual identity in the general population).^15^ Variations in these thresholds were however observed by age, which is not surprising as older people have had more time to engage in partnerships. Differences were also evident according to whether participants reported sexual health clinic attendance, which we used as a proxy for clinical populations. Different thresholds are therefore needed, and a practical algorithm developed, tested and evaluated, if this typology is to be used in the clinical context. While beyond the scope of this paper, this is an essential next step to ensure the typology's utility and validity.

A further strength of our partnership typology is that it requires data on just two items: partnership duration and the perceived likelihood of sex again with the partner, to differentiate between four types of partnership, at least in the British population. Furthermore, partnership duration can be measured simply as the number of months between first sex and most recent sex, and does not require exact dates or taking account of any breaks in the partnership, both of which are likely to be prone to recall bias. Ease of reporting is an attractive feature for patients, survey participants, researchers and clinicians alike. Furthermore, the question asked in Natsal-3 about the likelihood of having sex again was shown in cognitive interviewing to be generally easy to answer and perceived as inoffensive.^17^

In a clinical context, our partnership typology is timely, as sexual and public health practice move towards more detailed reporting of sexual risk and PN outcomes.^8^ Our analyses show that improving our understanding of STI risk requires ascertaining the type(s) of partner(s) and not just the number of partners someone has. This is particularly important as a large proportion of the population only has one partner in a year;^15^ so, even though STI prevalence is relatively low among those with fewer partners,^11^ they account for a large proportion of the burden of some STIs (eg, Chlamydia).^11^ We observed interesting gender differences in reported STI diagnosis by partnership type among those with just one partner; men for whom this partner was ‘now steady’ or ‘casual’ were more likely to report STI diagnoses than those cohabiting, but there was no difference in prevalence or adjusted odds ratio by partnership type among women with one partner. This finding emphasises the need to take account an individual's sexual behaviour and that of their partner(s), for example, the number and types of partners someone's partner has had. However, data are seldom collected directly from partners, and studies that have done so have shown that individuals do not always accurately judge their partner's sexual behaviour.^18–20^ Obtaining robust data on partners' behaviours—either directly or indirectly—therefore remains a challenge despite their epidemiological importance.

As the public health purse shrinks,^21^ more effective targeting of health promotion messaging and PN is required. Previous research has shown that there could be considerable public health gain by improving PN outcomes for casual partners, as those with casual partners tend to have more partners and thus a greater potential for onward transmission.^2^ An objective way of differentiating between different types of partners is thus required if this is to be achieved, and our typology is the first step in filling this evidence gap. The relevance and utility of our partnership typology clearly extend beyond reducing STI transmission to the broader sexual health arena.^22^ Its implications also apply beyond the clinical context to any, and all, scientific research that seeks to distinguish between different types of sexual partnership where definitions of partnership type are rarely provided.^6^ ^7^ ^11^ We hope our initial focus therefore acts to stimulate interest and provides an impetus for furthering our work.

As far as we are aware, this is the first study that has been attempted to use an evidence-based approach to empirically define partnership type in the context of a general population. Future studies should examine the extent to which partnership duration and likelihood of sex again, and the categories identified here, can be used to differentiate between different types of partnership in other settings. In this respect, the first stage of the LUSTRUM (‘Limiting Undetected Sexual Transmission to RedUce Morbidity’) study, a National Institute for Health Research Applied Research Programme (reference number: RP-PG-0614-20009), will look at these issues with clinicians and patients to ascertain the extent to which our partnership typology relates to both lay and professional understanding. This is likely to be particularly important in the context of non-cohabiting partnerships. For example, our label of ‘casual’ corresponds to just three PPTs; yet, a much wider array of casual partnership types has been identified.^23^ In addition, where cohabiting and ex-steady partnerships are considered as more ‘fluid’ terms, for example, ‘living apart together’ couples,^24^ ^25^ then our empirical-based categorisation will also be helpful. This reflects how cultural and temporal differences may mean that a universally agreed and accepted partnership typology is not possible; nonetheless, we hope our work will be valued for providing an objective method to differentiate between different types of partners.

**Key messages**

- Sexual partnership type is poorly and inconsistently recorded in clinical practice and research, limiting the effectiveness of interventions, such as partner notification.
- We developed an empirical-based method to categorise partnership type using data from >14 000 partnerships reported in Britain's third National Survey of Sexual Attitudes and Lifestyles.
- We use this method to demonstrate how reporting STI diagnoses varies according to the type(s) of partnership reported *over and above* reported partner numbers.
- Our novel, evidence-based typology provides an objective method for clinicians and epidemiologists to measure sexual partnership type, overcoming ambiguity and maximising health gain.
