# Supplementary material for: How can we objectively categorise partnership type? A novel classification of population survey data to inform epidemiological research and clinical practice
Source: Sex Transm Infect. 2016 Aug 17;93(2):129–36. doi: 10.1136/sextrans-2016-052646 (PMC5339562; doi:10.1136/sextrans-2016-052646)
Supplement: Supplementary tables [file sextrans-2016-052646supp_tables.pdf]

Supplementary Table 1: Partnerships where sex occurred on only one occasion (to date) by Summary Partnership Type, Partnership Progression Type, and participant's gender

		Denominators[1] (unweighted, weighted)	**% (95% CI) of partnerships where sex was reported only once**		
			**All**	**Men's partnerships**	**Women's partnerships**
**All partnerships**		***14048, 13387***	**11.0% (10.4%-11.6%)**	**13.3% (12.4%-14.3%)**	**8.4% (7.7%-9.0%)**
**Summary Partnership Type: Cohabiting**		***6493, 8008***	**1.9% (1.6%-2.3%)**	**2.1% (1.6%-2.7%)**	**1.7% (1.3%-2.2%)**
Partnership Progression Type:					
Living together	Living together	*978, 1247*	12.1% (10.0%-14.5%)	14.0% (10.7%-18.2%)	10.3% (8.1%-13.2%)
**Summary Partnership Type: Now steady**		***3816, 2635***	**4.6% (3.9%-5.4%)**	**5.4% (4.0%-6.4%)**	**4.1% (3.3%-5.2%)**
Partnership Progression Type:					
Steady	Steady	*1785, 1247*	9.7% (8.3%-11.4%)	11.1% (8.8%-14.0%)	8.4% (6.7%-10.4%)
**Summary Partnership Type: Casual**		***3111, 2339***	**51.1% (49.1%-53.1%)**	**53.7% (50.9%-56.4%)**	**46.7% (43.9%-49.6%)**
Partnership Progression Type:					
Known a while	Known a while	*1662, 1263*	38.5% (35.9%-41.1%)	38.5% (34.9%-42.3%)	38.4% (34.8%-42.2%)
Recently met	Recently met	*817, 662*	57.2% (53.4%-61.0%)	59.5% (54.4%-64.3%)	53.1% (47.2%-58.9%)
Just met	Just met	*444, 362*	91.2% (87.8%-93.7%)	91.1% (87.0%-94.0%)	91.7% (84.6%-95.7%)
**Summary Partnership Type: Ex-steady**		***449, 245***	**N/A**	**N/A**	**N/A**

Note for Supplementary Table 1:

[1] Denominators correspond to the number of partnerships (not participants).

Supplementary Table 2: Distribution of different combinations of Summary Partnership Type(s) in the past year, by number of partners reported in detail and participant's gender

Number of partners reported in detail:	*Denominators[1](unweighted, weighted)* Combination of Summary Partnership Type(s):	**All participants** *11034, 11177* **% (95% CI)**	**Men** *4632, 5701* **% (95% CI)**	**Women** *6402, 5476* **% (95% CI)**
**1 partner**				
	1 cohabiting	67.5% (66.5-68.5)	65.2% (63.6-66.7)	69.9% (68.6-71.1)
	1 now steady	13.4% (12.8-14.0)	12.6% (11.7-13.5)	14.2% (13.3-15.1)
	1 ex-steady	1.3% (1.1-1.5)	1.0% (0.7-1.3)	1.7% (1.4-2.0)
	1 casual	3.7% (3.4-4.1)	4.8% (4.2-5.5)	2.6% (2.2-3.0)
**2 partners**				
	Both cohabiting	0.3% (0.2-0.5)	0.4% (0.2-0.7)	0.2% (0.1-0.4)
	1 cohabiting & 1 now steady	0.7% (0.5-0.9)	0.8% (0.5-1.3)	0.6% (0.4-0.8)
	1 cohabiting & 1 ex-steady	0.1% (0.1-0.3)	0.2% (0.1-0.5)	0.1% (<0.1-0.2)
	1 cohabiting & 1 casual	1.6% (1.3-1.9)	2.1% (1.6-2.6)	1.0% (0.8-1.4)
	Both now steady	1.1% (0.9-1.3)	1.1% (0.8-1.4)	1.1% (0.9-1.4)
	1 now steady & 1 ex-steady	0.4% (0.3-0.5)	0.4% (0.2-0.6)	0.3% (0.2-0.5)
	1 now steady & 1 casual	2.5% (2.3-2.9)	3.0% (2.5-3.5)	2.1% (1.8-2.5)
	Both ex-steady	<0.1% (<0.1-0.1)	0.1% (<0.1-0.1)	<0.1% (<0.1-0.1)
	1 ex-steady & 1 casual	0.5% (0.4-0.7)	0.5% (0.3-0.7)	0.5% (0.4-0.7)
	Both casual	1.2% (1.0-1.4)	1.5% (1.2-1.9)	0.8% (0.6-1.1)
**3 partners**				
	All 3 cohabiting	<0.1% (<0.1-0.2)	<0.1% (<0.1-0.1)	<0.1% (<0.1-0.3)
	All 3 now steady	0.2% (0.1-0.2)	0.2% (0.1-0.3)	0.1% (0.1-0.2)
	All 3 casual	1.3% (1.1-1.5)	1.7% (1.3-2.0)	0.9% (0.7-1.1)
	2 cohabiting & 1 now steady	<0.1% (<0.1-0.1)	<0.1% (<0.1-0.2)	0
	2 cohabiting & 1 ex-steady	<0.1% (<0.1-0.1)	<0.1% (<0.1-0.2)	0
	2 cohabiting & 1 casual	<0.1% (<0.1-0.1)	<0.1% (<0.1-0.1)	<0.1% (<0.1-0.1)
	2 now steady & 1 cohabiting	0.1% (<0.1-0.1)	<0.1% (<0.1-0.2)	0.1% (<0.1-<0.1)
	1 cohabiting, 1 now steady & 1 ex-steady	<0.1% (<0.1-0.1)	<0.1% (<0.1-0.2)	0.1% (<0.1-0.2)
	1 cohabiting, 1 now steady & 1 casual	0.3% (0.2-0.4)	0.2% (0.1-0.5)	0.3% (0.2-0.5)
	1 cohabiting, 1 ex-steady & 1 casual	0.1% (<0.1-0.1)	0.1% (<0.1-0.2)	<0.1% (<0.1-0.1)
	2 casual & 1 cohabiting	0.5% (0.4-0.7)	0.7% (0.4-1.1)	0.3% (0.2-0.5)
	2 now steady & 1 ex-steady	0.1% (<0.1-0.1)	<0.1% (<0.1-0.1)	0.1% (0.1-0.2)
	2 now steady & 1 casual	0.7% (0.6-0.9)	0.8% (0.6-1.1)	0.7% (0.5-0.9)
	2 ex-steady & 1 now steady	<0.1% (<0.1-0.1)	<0.1% (<0.1-0.1)	<0.1% (<0.1-0.1)
	1 now steady, 1 ex-steady & 1 casual	0.4% (0.3-0.6)	0.5% (0.3-0.7)	0.4% (0.3-0.5)
	2 casual & 1 now steady	1.5% (1.3-1.8)	1.7% (1.4-2.1)	1.4% (1.1-1.7)
	2 ex-steady & 1 casual	0.1% (<0.1-0.1)	<0.1% (<0.1-0.1)	0.1% (<0.1-0.2)
	2 casual & 1 ex-steady	0.4% (0.3-0.5)	0.4% (0.3-0.6)	0.4% (0.3-0.6)
	Total:	*100%*	*100%*	*100%*

Note for Supplementary Table 2:
[1] Denominators correspond to the number of participants (not partnerships).

Supplementary Table 3A: Variations in reporting any STI diagnosis by combination of Summary Partnership Type(s), all in the past year: Men

		**% reporting STI diagnosis (95% CI)**	**Crude OR (95% CI)**	**OR adjusted for age & partner numbers, past year (95% CI)**	*Denominators[1] unweighted/ weighted*
**All**		1.0% (0.7-1.3)	-	-	*4337, 5390*
**Number of partners reported in detail:**	**Combination of Summary Partnership Type(s):**				
**1 partner**					
	Cohabiting	0.4% (0.3-0.8)	1.00	1.00	*2354, 3687*
	Now steady	1.7% (0.9-3.2)	3.91 (1.69-9.04)	2.20 (0.80-6.07)	*849, 708*
	Ex-steady	0.0%	N/A	N/A	*70, 55*
	Casual	2.2% (0.9-5.3)	4.94 (1.67-14.6)	3.22 (1.01-10.3)	*300, 275*
**2 partners**					
	Cohabiting & casual	0.8% (0.1-5.6)	1.82 (0.23-14.2)	1.38 (0.17-10.8)	*82, 114*
	Both now steady	1.8% (0.2-11.6)	4.03 (0.51-31.6)	2.12 (0.27-16.5)	*74, 61*
	Now steady & casual	2.1% (0.8-5.4)	4.80 (1.55-14.9)	2.22 (0.68-7.21)	*200, 169*
	Both casual	3.8% (1.5-9.6)	8.88 (2.86-27.6)	4.27 (1.18-15.5)	*115, 86*
**3 partners**					
	All casual	3.2% (1.1-8.6)	7.33 (2.23-24.1)	2.71 (0.72-10.2)	*114, 93*
	2 now steady & 1 casual	0.9% (0.1-6.3)	2.06 (0.26-16.2)	0.88 (0.11-7.10)	*59, 45*
	2 casual & 1 now steady	6.0% (2.5-13.5)	14.2 (4.96-40.7)	6.03 (2.01-18.1)	*120, 98*

Note for Supplementary Table 3A:
[1] Denominators correspond to the number of participants (not partnerships).

Supplementary Table 3B: Variations in reporting any STI diagnosis by combination of Summary Partnership Type(s), all in the past year: Women

		% reporting STI diagnosis (95% CI)	Crude OR (95% CI)	OR adjusted for age & partner numbers, past year (95% CI)	*Denominators[1] unweighted/ weighted*
**All**		1.0% (0.7%-1.2%)	-		*6119, 5309*
**Number of partners reported in detail:**	**Combination of Summary Partnership Type(s):**				
**1 partner**					
	Cohabiting	0.3% (0.2%-0.5%)	1.00	1.00	*3646, 3808*
	Now steady	1.8% (1.1%-2.9%)	6.31 (3.02-13.2)	2.18 (0.96-4.96)	*1265, 775*
	Ex-steady	0.0%	N/A	N/A	*157, 92*
	Casual	0.4% (0.1%-1.8%)	1.58 (0.35-7.03)	0.78 (0.17-3.60)	*218, 142*
**2 partners**					
	Cohabiting & now steady	6.1% (2.1%-16.5%)	22.8 (6.66-78.1)	8.10 (2.37-27.7)	*54, 31*
	Cohabiting & casual	3.1% (0.8%-11.9%)	11.4 (2.48-52.5)	6.73 (1.34-33.7)	*74, 57*
	Both now steady	3.4% (1.2%-9.4%)	12.6 (3.84-41.5)	3.49 (1.08-11.3)	*113, 60*
	Now steady & casual	4.2% (2.2%-8.2%)	15.7 (6.56-37.6)	3.93 (1.56-9.93)	*206, 115*
	Ex-steady & casual	6.3% (2.0%-18.1%)	24.0 (6.78-84.9)	7.78 (2.27-26.7)	*55, 29*
	Both casual	4.8% (1.3%-15.7%)	17.8 (4.36-73.1)	5.71 (1.39-23.5)	*63, 44*
**3 partners**					
	All casual	8.7% (3.8%-18.9%)	33.8 (12.0-95.3)	6.52 (2.05-20.7)	*77, 47*
	2 now steady & 1 casual	5.0% (1.7%-13.4%)	18.5 (5.54-61.9)	3.11 (0.72-13.4)	*62, 36*
	2 casual & 1 now steady	6.8% (3.2%-13.8%)	25.9 (10.1-66.2)	5.43 (1.92-15.3)	*129, 74*

Note for Supplementary Table 3B:
[1] Denominators correspond to the number of participants (not partnerships).
